# Young bone marrow transplantation prevents aging‐related muscle atrophy in a senescence‐accelerated mouse prone 10 model

**DOI:** 10.1002/jcsm.13058

**Published:** 2022-09-04

**Authors:** Aiko Inoue, Limei Piao, Xueling Yue, Zhe Huang, Lina Hu, Hongxian Wu, Xiangkun Meng, Wenhu Xu, Chenglin Yu, Takeshi Sasaki, Kohji Itakura, Hiroyuki Umegaki, Masafumi Kuzuya, Xian Wu Cheng

**Affiliations:** ^1^ Institute of Innovation for Future Society Nagoya University Graduate School of Medicine Nagoya Aichiken Japan; ^2^ Department of Community Healthcare and Geriatrics Nagoya University Graduate School of Medicine Nagoya Aichiken Japan; ^3^ Department of Cardiology and Hypertension, Jilin Provincial Key Laboratory of Stress and Cardiovascular Disease Yanbian University Hospital Yanji Jilin PR China; ^4^ Department of Human Cord Applied Cell Therapy Nagoya University Graduate School of Medicine Nagoya Aichiken Japan; ^5^ Department of Public Health Guilin Medical College Guilin Guangxi PR China; ^6^ Shanghai Institute of Cardiovascular Disease, Zhongshan Hospital Fudan University Shanghai PR China; ^7^ Department of Anatomy and Neuroscience Hamamatsu University School of Medicine Hamamatsu Shizuokaken Japan; ^8^ Division for Medical Research Engineering Nagoya University Graduate School of Medicine Nagoya Aichiken Japan

**Keywords:** Aging, Sarcopenia, Mouse model, Bone marrow transplantation, Muscle stem cell, SAMP10

## Abstract

**Background:**

Young bone marrow transplantation (YBMT) has been shown to stimulate vascular regeneration in pathological conditions, including ageing. Here, we investigated the benefits and mechanisms of the preventive effects of YBMT on loss of muscle mass and function in a senescence‐associated mouse prone 10 (SAMP10) model, with a special focus on the role of growth differentiation factor 11 (GDF‐11).

**Methods:**

Nine‐week‐old male SAMP10 mice were randomly assigned to a non‐YBMT group (*n* = 6) and a YBMT group (*n* = 7) that received the bone marrow of 8‐week‐old C57BL/6 mice.

**Results:**

Compared to the non‐YBMT mice, the YBMT mice showed the following significant increases (all P < 0.05 in 6–7 mice): endurance capacity (>61.3%); grip strength (>37.9%), percentage of slow myosin heavy chain fibres (>14.9–15.9%). The YBMT also increased the amounts of proteins or mRNAs for insulin receptor substrate 1, p‐Akt, p‐extracellular signal‐regulated protein kinase1/2, p‐mammalian target of rapamycin, Bcl‐2, peroxisom proliferator‐activated receptor‐γ coactivator (PGC‐1α), plus cytochrome c oxidase IV and the numbers of proliferating cells (n = 5–7, P < 0.05) and CD34+/integrin‐α7+ muscle stem cells (n = 5–6, P < 0.05). The YMBT significantly decreased the levels of gp91phox, caspase‐9 proteins and apoptotic cells (n = 5–7, P < 0.05) in both muscles; these beneficial changes were diminished by the blocking of GDF‐11 (n = 5–6, P < 0.05). An administration of mouse recombinant GDF‐11 improved the YBMT‐mediated muscle benefits (n = 5–6, P < 0.05). Cell therapy with young bone marrow from green fluorescent protein (GFP) transgenic mice exhibited GFP+ myofibres in aged muscle tissues.

**Conclusions:**

These findings suggest that YBMT can prevent muscle wasting and dysfunction by mitigating apoptosis and proliferation via a modulation of GDF‐11 signalling and mitochondrial dysfunction in SAMP10 mice.

## Introduction

The aging‐related loss of skeletal muscle mass and function, that is, sarcopenia, is associated with physical frailty and increased risks of chronic disease outcomes.^1^ The pathogenesis of sarcopenia involves multiple biological factors including apoptotic and inflammatory factors as well as an imbalance of protein synthesis and mitochondrial dysfunction in the muscles of aged humans and animals.^2^, ^3^, ^4^, ^5^ There have been several clinical and experimental studies of sarcopenia, which is present in many pathogeneses,^3^, ^4^, ^5^, ^6^ but the incidence of age‐related sarcopenia continues to increase, with no known cure. A clarification of the pathogenesis of sarcopenia is thus urgently needed, as is the identification of novel preventive and therapeutic targets for age‐dependent muscle degeneration.^7^

The generative potential of bone marrow‐derived mesenchymal stem/stromal cells (BM‐MSCs) is demonstrated by the ability of these cells to differentiate into diverse tissues and by their anti‐inflammatory and immunomodulatory properties via the cells' secretions of a variety of anti‐inflammatory cytokines and growth factors (for review, see references^8^, ^9^). Cell therapy with a local injection of autologous BM‐MSCs was reported to be an effective and safe way to promote the recovery of irradiation‐induced skeletal muscle injury, with no signs of long‐term degeneration.^10^ The co‐delivery of bone marrow (BM)‐derived mononuclear cells with minced muscle grafts was described as a promising treatment paradigm for volumetric muscle loss injury.^11^ BM‐MSC‐derived CXCL12 (C‐X‐C motif chemokine ligand 12) and osteopontin have been reported to contribute to muscle regeneration through STAT3 signalling in satellite cells called muscle stem/progenitor cells.^12^ CXCR4^+^ BM‐MSCs interaction with the satellite cells induced their myogenic commitments.^13^ Squecco and colleagues recently showed that BM‐MSC‐derived secretome as conditioned medium protects against an experimental skeletal muscle injury induced by *ex vivo* eccentric contraction.^14^ However, it has become clear that the number and function of BM‐derived stem cells are modified by the aging that limits the cell therapy‐mediated muscle benefit in aged animals.^15^, ^16^, ^17^

Growth differentiation factor‐11 (GDF‐11), a member of the transforming growth factor‐beta (TGF‐β) superfamily, regulates various cellular functions.^18^ Observations from clinical and laboratory investigations indicated that aging is accompanied by a decline in the level of circulating GDF‐11.^19^, ^20^, ^21^ In heterochronic parabiosis experiments, GDF‐11 played a role as a rejuvenation factor to reverse age‐related cardiac hypertrophy and skeletal muscle dysfunction in mice.^21^, ^22^ GDF‐11 was also shown to exert rejuvenating benefits on the central nervous system of mice by rectifying age‐related cerebral vascular remodelling and neuronal stem cell dysfunction.^23^ However, Egerman and colleagues reported that GDF‐11 increased with age and inhibited skeletal muscle regeneration in rat and human sera^24^; it was also reported that the circulating GDF‐11 levels showed no decline with age or correlation with aging in a healthy population.^25^, ^26^ Further research is thus needed to clarify these discrepancies.

Here, we report that in a senescence‐accelerated mouse prone 10 (SAMP10) model, young bone marrow transplantation (YBMT) relieved the loss of muscle mass and dysfunction via modulations of the protein anabolism, apoptosis, and mitochondrial biogenesis that are mediated by elevated circulating GDF‐11 levels.

## Materials and methods

### Mice

The present animal studies using young (24‐week‐old) and old (40‐week‐old) male SAMP10 mice (SAMP10/TaSlc, Japan SLC, Hamamatsu, Japan) and young (8‐week‐old) and green fluorescent protein (GFP)‐transgenic mice (Japan SLC) were approved by the Institutional Animal Care and Use Committee at Nagoya University and were conducted in accord with the US National Institutes of Health (NIH) Guide for the Care and Use of Laboratory Animals. The mice were housed in a room with a controlled 12‐h light–dark cycle and temperature (23 ± 2°C), with ad libitum access to food and water.

### Endurance measurement

The running endurance capacity of the mice was evaluated with the use of a motorized rodent treadmill (S‐CON MINI‐Z: Tokyo Engineering, Tokyo) as described.^27^ A mouse was deemed to be fatigued when it was no longer able to continue to run on the treadmill. We evaluated the grip strength of the mice by using a small‐animal grip strength meter (Columbus Co., Largo, FL, USA). When the forelimbs of a mouse whose tail was pulled horizontally by an examiner's hand were no longer able to grasp the strength meter, the suggested force was considered the maximum grip strength. The grip strength value was evaluated and averaged over five calculations for each mouse. On the last day of the experiment, the mice were euthanized by an overdose of sodium pentobarbital, and the respective isolated muscles (gastrocnemius and soleus) were immediately subjected to the biochemical and morphological analyses.

### Bone marrow transplantation

Young bone marrow transplantation was performed with a direct injection of the donor BM (2 × 10^7^ per mouse) into the BM cavities of both tibias of irradiated mice.^28^ We investigated whether YBMT would exert preventive effects on muscle wasting in young SAMP10 mice. Young (24‐week‐old) SAMP10 mice (*n* = 24) received BM from 8‐week‐old C57BL/6 mice. Age‐matched SAMP10 mice without YBMT were used as the control (non‐YBMT) group. The mice were sacrificed at 40 weeks of age. For the determination of the contribution of BM‐MSCs to muscle regeneration, 8‐week‐old male GFP‐transgenic mice^15^ were also used for YBMT.

For the experiment involving the blocking of GDF‐11, SAMP10 mice that had undergone YBMT were randomly assigned to one of two groups that were weekly injected subcutaneously with either mouse control IgG (450 μg/kg, Abcam, Cambridge, MA, USA) or a neutralizing antibody against GDF‐11 (NGDF‐11, 450 μg/kg, R&D Systems, Minneapolis, MN, USA) for 3 months. For the experiment concerning supplementation with GDF‐11, SAMP10 mice that had undergone YBMT were randomly assigned to one of two groups weekly injected subcutaneously with either saline alone (vehicle, 50 μL/time) or recombinant GDF‐11 (rGDF, 450 μg/kg, R&D Systems). The mice were sacrificed at 36 weeks of age.

### Morphometric and immunohistological analyses

First, the collected skeletal tissue sections (4‐μm thickness) were stained with haematoxylin–eosin (H&E) using standard protocols. For the immunohistological analysis, the corresponding skeletal muscle 4‐μm‐thick sections were treated with a mouse monoclonal antibody (mAb) against proliferating cell nuclear antigen (PCNA: 1:00, Abcam), a mouse mAb against mouse Pax7 (sc‐81975, 1:40, Santa Cruz Biotechnology, Santa Cruz, CA, USA), a rat mAb against macrophages (Mac 3; 1:40, BD Pharmingen, San Diego, CA, USA), and a rabbit polyclonal antibody (pAb) against slow myosin heavy chain (MHL; 1:100, Abcam). The sections were then visualized with an ABC substrate kit (Vector Laboratories, Burlingame, CA, USA). The muscle fibre size and crown‐like structures on the stained slides were analysed under ×200 magnification in a blinded fashion by two independent investigators. The area per muscle fibre was measured in three randomly chosen microscopic fields from six different sections in each tissue block and averaged for each animal. The skeletal muscle fibre sizes were calculated using Win ROOF version 5.02 (Mitani, Fukui, Japan).

Double immunofluorescence was conducted with a goat pAb against integrin‐α7 (1:100; Santa Cruz Biotechnology), a rabbit mAb against CD34 (1:100; Abcam), a mouse mAb against desmin (1:100; Dako, Carpinteria, CA, USA), and a rabbit pAb against laminin‐5 (Bioss Antibodies, Woburn, MA, USA). The results were visualized using Zenon rabbit and mouse IgG labelling kits (1:200; Molecular Probes, Eugene, OR, USA).^4^ For negative control staining, the primary antibodies were replaced with nonimmune immunoglobulin G or mouse IgG or Zenon‐labelled rabbit IgG.

### Biochemical analysis and enzyme‐linked immunosorbent assay

The plasma levels of blood urea nitrogen (BUN), high‐density lipoprotein (HDL), low‐density lipoprotein (LDL), glucose, creatinine, and triglyceride in the mice were determined by the Nagoya University Clinical Research Laboratory. The levels of plasma GDF‐11, basic‐fibroblast growth factor (bFGF), tumour necrosis factor‐alpha (TNF‐α), vascular endothelial growth factor (VEGF), and adiponectin in the experimental groups (*n* = 6–9 for each group) were measured by a commercially available enzyme‐linked immunosorbent assay (ELISA) kit (R&D Systems) according to the manufacturer's instructions.

### Western blot analysis

Tissue samples obtained from 40‐week‐old mice were homogenized in lysis buffer in the presence of 20 mM of Tris‐HCl (pH 8.0), 150 mM of NaCl, 1 mM of sodium orthovanadate, 1% Nonidet P‐40, 0.5% deoxycholic acid, and protease inhibitor mixture (Sigma‐Aldrich, St. Louis, MO, USA). The protein contents were studied by the Bradford method. Protein was extracted using a RIPA lysis buffer and western blotted against antibodies (1:1000 for each antibody) for total Smad2, phospho‐Samd2 (p‐Samd2), total Samd3, phospho‐Samd3 (p‐Samd3), total Akt, phospho‐Akt (p‐Akt), total Erk1/2, p‐extracellular signal‐regulated kinase1/2 (p‐Erk1/2), total mTOR, phospho‐mammalian target of rapamycin (p‐mTOR), p38 mitogen‐activated protein kinase (p38MAPK), phospho‐p38MAPK (p‐p38MAPK), Bcl‐2, caspase‐9, Bcl‐XL (Cell Signaling Technology, Beverly, MA, USA), myogenin, gp91phox, insulin receptor substrate‐1 (IRS‐1) (Santa Cruz Biotechnology), and as the loading control, glyceraldehyde 3‐phosphate dehydrogenase (GAPDH) (Sigma‐Aldrich).

### Quantitative polymerase chain reaction

Total RNA isolation, reverse‐transcription, and quantitative polymerase chain reaction (PCR) were performed as described.^27^ Quantitative gene expression was performed using the ABI 7300 real‐time PCR system with Universal PCR Master Mix (Applied Biosystems, Foster City, CA, USA). All gene expressions were measured in triplicate. Supporting Information, *Table* *S1* provides the sequences of the primers for cytochrome *c* oxidase (COX)‐III, COX‐IV (also called COX5B), glucose transporter‐4 (GLUT‐4), peroxisome proliferator‐activated receptor‐γ coactivator‐1α (PGC‐1α), and PGC‐1β genes. All target gene expressions were normalized to that of GAPDH.

### Electron microscopy analysis of myocardial mitochondria

Electron microscopy was performed as described.^29^ Muscle samples were cut into approximately 1‐mm^3^ pieces and fixed for 24 h with 2% glutaraldehyde in 0.16 M of phosphate‐buffered saline (PBS) (pH 7.2) and then for 1 h with 1% osmium tetroxide. The fixed tissues were dehydrated in a graded series of ethanol solutions before being exposed to propylene oxide and embedded in Epon. Sections were cut at 60‐ to 70‐nm thickness, stained with uranyl acetate and lead citrate, and observed with a JEM‐1400 transmission electron microscope (JEOL, Tokyo) operating at 100 kV. We determined the mitochondrial number and size at ×15 000 magnification by counting the corresponding number of pixels with the use of Adobe Photoshop CS5 software. A total of 60–80 mitochondrial cross‐sectional areas from six sections were measured for each mouse, and histograms were generated separately for each experimental group.

### Superoxide production analysis

We evaluated the nicotinamide adenine dinucleotide phosphate (NADPH)‐dependent superoxide (O_2_^−^) production by using total homogenates of the fresh skeletal muscle tissue with the use of a lucigenin‐based enhanced chemiluminescence assay, as described.^30^ A low lucigenin concentration (5 μmol/L) was used to minimize the artefactual O_2_^−^ production attributable to redox cycling. Briefly, 50 μg of homogenate protein diluted in 1 mL of lysis buffer (20 mmol/L of Tris‐HCl, 150 mmol/L of NaCl, 1 mmol of EDTA, 1 mmol/L of EGTA, and 1% Triton X‐100; pH 7.5) was transferred to an assay tube, and NADPH and dark‐adapted lucigenin were added to final concentrations of 100 and 5 μmol/L, respectively, immediately before the measurement of chemiluminescence. The chemiluminescence signal was sampled every 60 s for 12 min with a tube luminometer (20/20; Turner Designs, San Jose, CA, USA), and the respective background counts were subtracted from the experimental values. All of the assays were performed in triplicate.

### Isolation of bone marrow‐derived integrin α7^+^ stem cells

Bone marrow‐derived cells were obtained from the experimental groups of SAMP10 mice (*n* = 5 per group). Following the isolation of lineage^−^ mononuclear cells, BM‐derived integrin‐α7^+^ cells were obtained by using integrin‐α7 MicroBeads and magnetic‐activated cell sorting (MACS) (Miltenyi Biotec, Bergisch Gladbach, Germany). Integrin‐α7^+^ BM cells were >90% positive for integrin‐α7^+^. These cells expressed muscle stem cell (MuSC) surface markers such as CD34 and integrin α7.^31^ After the integrin‐α7^+^ cells were cultured on fibronectin‐coated dishes (Wako, Osaka, Japan) in Dulbecco's modified Eagle medium (DMEM) and 2% foetal bovine serum (FBS) for 7 days, the cells were applied to the cellular assay.

### Terminal deoxynucleotidyl transferase‐mediated dUTP nick end labelling staining

After the attachment of BM‐derived integrin‐α7^+^ cells (cell density of 2 × 10^4^ cells/mL) to coverslips precoated with gelatin, the cells were cultured in serum‐free DMEM containing 250 μM of H_2_O_2_ for 24 h and then subjected to terminal deoxynucleotidyl transferase‐mediated dUTP nick end labelling (TUNEL) staining.^4^ The apoptotic cells in the muscle tissues were also studied by TUNEL staining.

### Immunocytofluorescence

After the attachment of BM‐derived integrin‐α7^+^ cells (cell density of 2 × 10^4^ cells/mL) to coverslips precoated with gelatin, the cells were fixed for 10 min with 8% paraformaldehyde and washed with 1% glycerol. Following blocking with 0.1% bovine serum albumin (BSA)/PBS, the cells were studied using immunofluorescence.^4^

### Statistical analyses

The resulting data are presented as the mean ± standard error of the mean (SEM). Student's *t‐*tests (for comparisons of two groups) or a one‐way analysis of variance (ANOVA) (for comparisons of three or more groups) followed by Tukey's post hoc tests were used for the statistical analyses. Probability (*P*)‐values < 0.05 were considered significant.

## Results

Senescence‐accelerated mouse prone 10 mice underwent YBMT at 24 weeks of age. As seen in *Table* *S2*, with the exception of HDL, there were no significant differences in the levels of T‐cho, LDL, triglyceride, glucose, BUN, or creatinine between the non‐YBMT and YBMT groups at 40 weeks.

### Young bone marrow transplantation ameliorated muscle dysfunction and morphological changes

**Figure 1:**
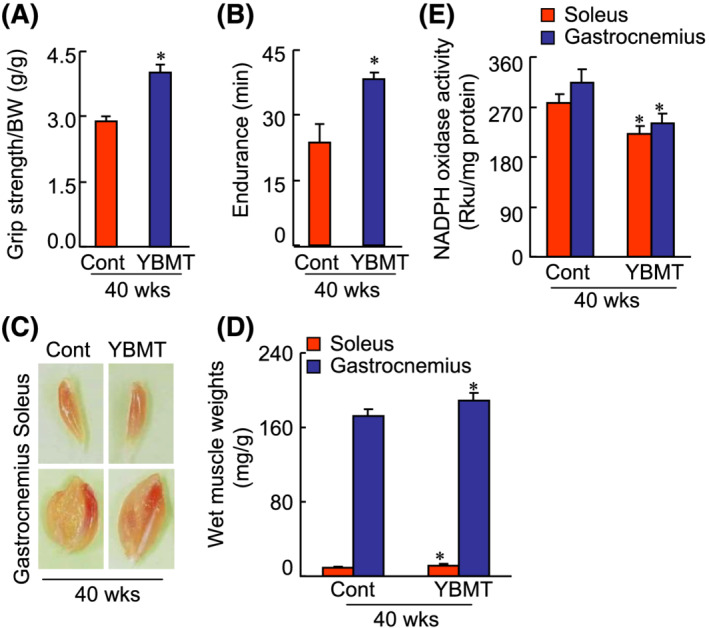
The effects of young bone marrow transplantation (YBMT) on the muscle mass and muscle performance of senescence‐accelerated mouse prone 10 (SAMP10) mice at 4 months post‐transplantation. (*A* and *B*) Grip strength/body weight (BW) and endurance were recorded in the control (Cont) and YBMT groups at 40 weeks of age. (*C* and *D*) Representative photos of soleus and gastrocnemius muscles. The weights of soleus and gastrocnemius muscles in the 40‐week‐old mice were calculated in both groups. (*E*) Nicotinamide adenine dinucleotide phosphate (NADPH) oxidase activity in homogenates of the skeletal muscle tissue. Data are expressed as relative light units (RLU) per milligram of protein. Data are means ± standard error of the mean (SEM) (*n* = 6–7). *P < 0.05 vs. the corresponding control groups by Student's *t*‐test or one‐way analysis of variance (ANOVA) followed by Tukey's post hoc tests. Scale bars: 50 μm.

**Figure 2:**
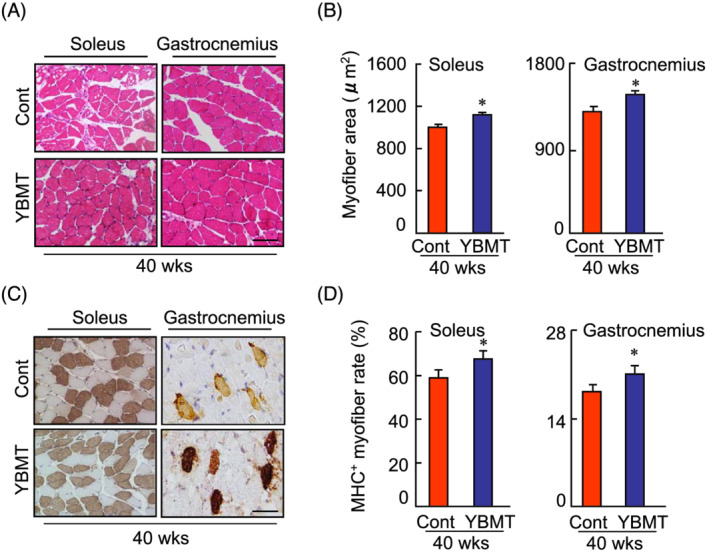
The effects of young bone marrow transplantation (YBMT) on myofibre size and the slow myosin heavy chain (MHC) rate in the muscle tissues of senescence‐accelerated mouse prone 10 (SAMP10) mice at 4 months post‐transplantation. (*A* and *B*) Representative haematoxylin–eosin (H&E) staining and quantitative data show the myofibre size of gastrocnemius and soleus muscles of control (Cont) and YBMT mice. (*C*) Representative MHC staining images used to calculate the numbers of MHC^+^ myofibres in the soleus and gastrocnemius of both groups. (*D*) Quantitative data showing the ratios of the MHC^+^ myofibres to total myofibres in both muscles. Data are mean ± standard error of the mean (SEM) (*n* = 6–7 per group). *P < 0.05, Student's *t*‐test. Scale bars: 50 μm.

Senescence‐accelerated mouse prone 10 mice are a model of aging, muscle atrophy, and dysfunction.^4^ We investigated the impact of YBMT on the age‐associated losses of muscle mass and function in SAMP10 mice at 16 weeks after the interventions, and we observed no significant difference in body weight between the YBMT and non‐YBMT experimental groups (data not shown). As seen in *Figure* 1A and 1B, YBMT relieved the impaired running endurance capacity and grip strength. YBMT was also followed by increased muscle mass (*Figure* 1C and 1D) and increased myofibre size as well as an increased slow major histocompatibility complex (MHC)‐positive myofibre rate (*Figure* 2), suggesting that the cellular morphological changes are responsible for the amelioration of muscle dysfunction in YBMT mice. On the other hand, NADPH oxidase assay revealed that the levels of superoxide production were lower in YBMT mice than that of the non‐YBMT mice (*Figure* 1E).

### Young bone marrow transplantation prevented cell loss and enhanced the muscle regeneration

**Figure 3:**
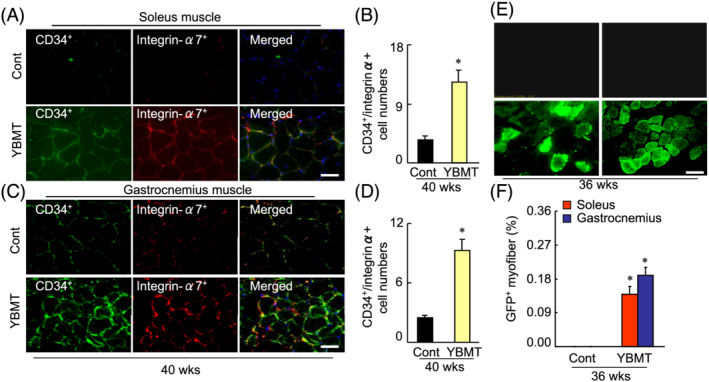
Young bone marrow transplantation (YBMT) increased the numbers of CD34^+^/integrin‐α7^+^ cells in the soleus and gastrocnemius muscles of senescence‐accelerated mouse prone 10 (SAMP10) mice at 40 weeks of age. (*A–D*) Representative images and combined quantitative data for muscle stem cells (MuSCs) in both muscles identified by double immunofluorescence staining with a rabbit monoclonal antibody (mAb) against CD34 and a goat polyclonal antibody (pAb) against integrin‐α7. (*E* and *F*) Following cell therapy with the bone marrow of green fluorescent protein (GFP)‐transgenic mice, both skeletal muscles were applied to a confocal microscopy analysis. Quantitative data show the percentages of GFP^+^ myofibres in the soleus and gastrocnemius muscles. Data are mean ± standard error of the mean (SEM) (*n* = 5–6). *P < 0.05 vs. the corresponding control groups by one‐way analysis of variance (ANOVA) followed by Tukey's post hoc tests. Scale bars: 50 μm.

**Figure 4:**
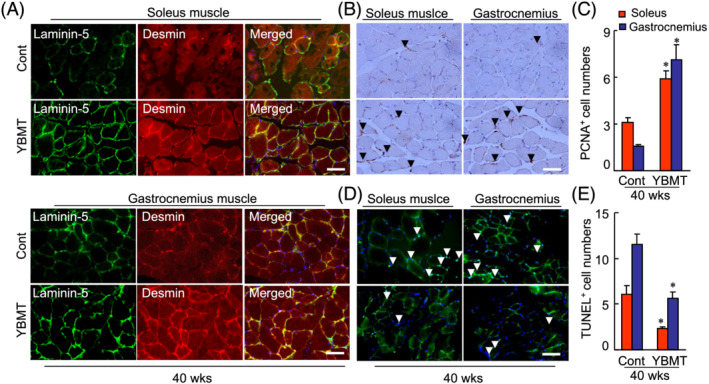
Young bone marrow transplantation (YBMT) improved the desmin expression and cell loss in the soleus and gastrocnemius of senescence‐accelerated mouse prone 10 (SAMP10) mice at 40 weeks of age. (*A*) Fluorescence staining of muscles with a laminin‐5 rabbit polyclonal antibody (pAb) (*green*) and a desmin monoclonal antibody (mAb) (*red*). (*B*) Representative proliferating cell nuclear antigen (PCNA) immunostaining with a mouse mAb used to assess the content of proliferated cells. (*C*) Quantitative data of PCNA^+^ cells in both muscles. (*D*) Representative terminal deoxynucleotidyl transferase‐mediated dUTP nick end labelling (TUNEL) staining used to assess the content of apoptotic cells. (*E*) Quantitative data of TUNEL^+^ cells in both muscle types. Data are mean ± standard error of the mean (SEM) (*n* = 5–7). *P < 0.05 vs. the corresponding control groups by one‐way analysis of variance (ANOVA) followed by Tukey's post hoc tests. *Arrowhead*: Related staining positive cells. Scale bars: 50 μm.

**Figure 5:**
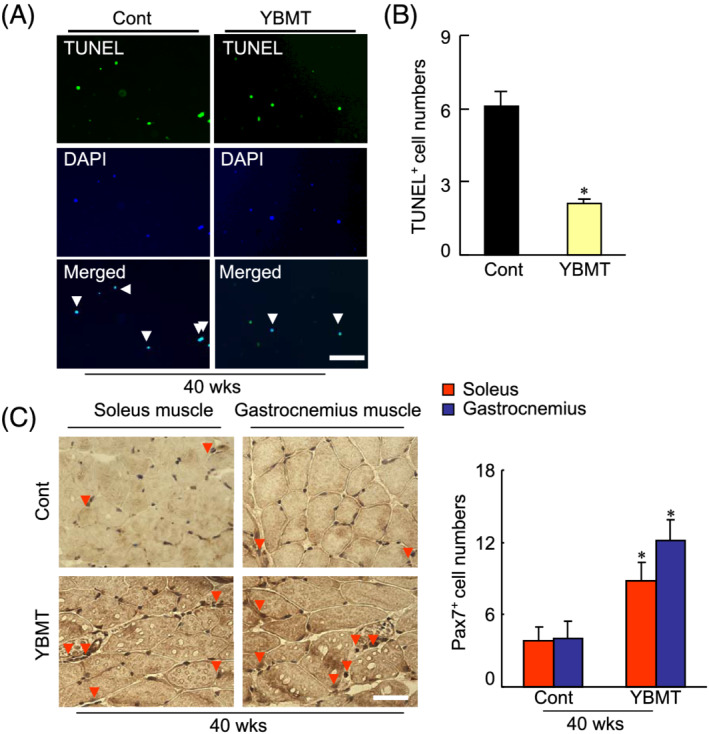
Young bone marrow transplantation (YBMT) improved bone‐derived integrin α^+^ cell apoptosis in the muscles of senescence‐accelerated mouse prone 10 (SAMP10) mice at 40 weeks of age. (*A* and *B*) Representative images of terminal deoxynucleotidyl transferase‐mediated dUTP nick end labelling (TUNEL) staining and quantitative data of integrin α^+^ cell apoptosis in the soleus and gastrocnemius muscles of SAMP10 mice at 40 weeks of age. (*C*) Representative images and combined quantitative data for Pax7^+^ cells in both muscles identified by immunostaining with a mouse monoclonal antibody (mAb) against Pax7. Data are means ± standard error of the mean (SEM) (*n* = 5–7). *P < 0.05 vs. the corresponding control groups by Student's *t*‐test or one‐way analysis of variance (ANOVA) followed by Tukey's post hoc tests. Scale bars: 50 μm.

Bone marrow‐derived MuSCs have been shown to contribute to skeletal muscle regeneration in several animal models of muscle disease.^10^, ^11^, ^12^, ^32^ To further examine the contribution of YBMT to the skeletal muscle regeneration process, we characterized MuSCs of the soleus and gastrocnemius muscles of 40‐week‐old mice by applying double immunofluorescence staining. The numbers of CD34^+^/integrin‐α^+^ MuSCs were higher in both soleus and gastrocnemius muscles of the YBMT mice compared with the control mice (*Figure* 3A–3D). The YBMT with BM from GFP‐transgenic mice demonstrated BM‐MSC homing and the differentiation of BM‐MSCs into skeletal muscle cells (*Figure* 3E and 3F). The use of double immunofluorescence revealed that compared with the muscles of control mice, the soleus and gastrocnemius muscles of the YBMT mice exhibited a diffuse and strong positive staining signal for desmin (*Figure* 4A), suggesting that BM‐MSCs might contribute to skeletal muscle regeneration. Aging has been shown to cause a harmful change in BM‐derived stem cell functions.^15^, ^17^, ^33^ As shown in *Figure* 5A and 5B, the numbers of TUNEL^+^ cells were lower in the BM‐derived integrin‐α^+^ cells of YBMT mice compared with the non‐YBMT mice. We observed that the numbers of Pax7^+^ cells were higher in the both muscles of the YBMT mice compared with the control mice (*Figure* 5C).

In addition, because the deterioration of muscle cell proliferation and the enhancement of apoptosis have seemed to be closely associated with aged muscle mass loss,^4^, ^5^ we evaluated the effects of YBMT on proliferation and apoptosis. The numbers of PCNA^+^ proliferating cells were higher and the numbers of TUNEL^+^ cells were lower in both the soleus and gastrocnemius muscles of the YBMT mice compared with the corresponding muscles of the control mice (*Figure* 4B–4E), indicating that the improvement of cell proliferation ability and apoptosis may contribute to the mitigation of the skeletal muscle mass loss in YBMT SAMP10 mice.

**Figure 6:**
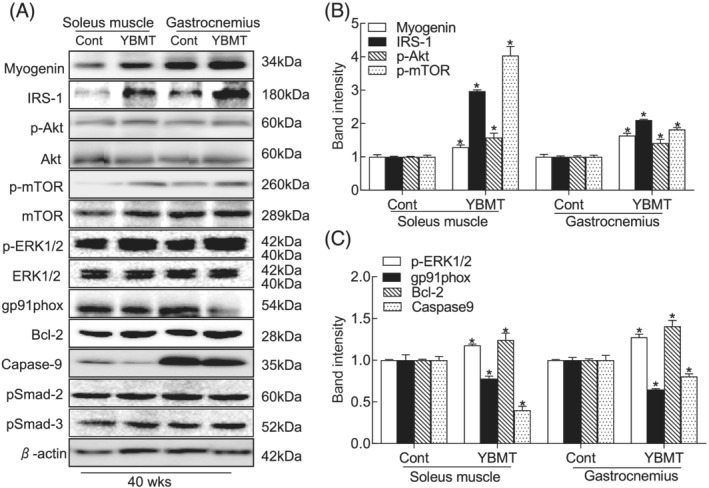
The effects of young bone marrow transplantation (YBMT) on targeted protein expression and phosphorylation in the muscles of senescence‐accelerated mouse prone 10 (SAMP10) mice at 40 weeks of age. (*A–C*) Representative immunoblot images and combined quantitative data of the expression levels of the investigated proteins (IRS1, Bcl‐2, caspase‐9, myogenin, and gp91phox) or phosphorylated proteins (p‐mTOR, p‐Erk1/2, and p‐Akt) in the soleus and gastrocnemius.. The expression level of each targeted protein was normalized with a western blot antibody to β‐actin. Data are mean ± standard error of the mean (SEM) (*n* = 3). *P < 0.05 vs. the corresponding control groups by one‐way analysis of variance (ANOVA) followed by Tukey's post hoc tests.

Next, to further examine the consequences of YBMT‐induced molecular changes, we used extracted muscle samples to study the oxidative stress‐related, protein anabolism‐related, and apoptosis‐related signalling pathways. The results indicated that YBMT elevated the levels of IRS‐1 protein, a key player in insulin growth signalling, in the soleus and gastrocnemius muscles (*Figure* 6). YBMT was also followed by elevated levels of myogenin, p‐Akt, p‐mTOR, and p‐Erk1/2 and reduced levels of gp91phox and caspase‐9 in both muscle tissues (*Figure* 6A–6C). These observations indicate that in SAMP10 mice, the YBMT‐mediated skeletal muscle protective response may be attributable to IRS‐1/Akt–mTOR signalling activation‐mediated protein anabolism, anti‐oxidative stress, and anti‐apoptosis. However, there were no significant differences between the control and YBMT mice in the levels of p‐Samd2 or p‐Samd3 in the soleus or gastrocnemius muscles.

### Young bone marrow transplantation mitigated muscle mitochondrial dysfunction

**Figure 7:**
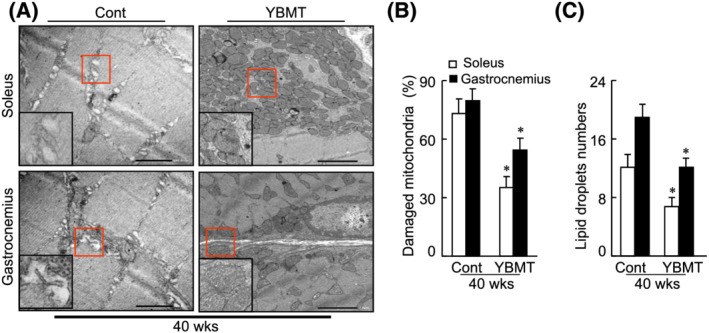
Young bone marrow transplantation (YBMT) ameliorated mitochondria damage and lipid droplet accumulation in the soleus and gastrocnemius muscles of senescence‐accelerated mouse prone 10 (SAMP10) mice at 40 weeks of age. (*A*) Representative electron microscopy images show a relatively preserved mitochondrial configuration as well as a small amount of lipid droplets. (*B* and *C*) Quantitative data of damaged mitochondrial numbers, the percentage of damaged mitochondria, and lipid droplet numbers. Data are mean ± standard error of the mean (SEM) (*n* = 4–5). *P < 0.05 vs. the corresponding control groups by one‐way analysis of variance (ANOVA) followed by Tukey's post hoc tests. Scale bars: 500 nm.

We further examined whether YBMT would influence the age‐associated mitochondrial morphological changes and dysfunction. Our electron microscopy observations revealed severe degeneration in the cristae of mitochondria in both the soleus and gastrocnemius muscles of the SAMP10 mice (*Figure* 7A). As shown in *Figure* 7B and 7C, the numbers of damaged mitochondria and the lipid‐like drops were lower in the muscles of YBMT mice compared with those of the non‐YBMT mice, indicating that YBMT ameliorated skeletal muscle mitochondrial biogenesis and degeneration in these SAMP10 mice. The results also indicate that YBMT mitigated the damage to mitochondria and the accumulation of lipid droplets (*Figure* 7B and 7C). Likewise, the quantitative PCR results showed that the expression levels of COX4 mRNA, a key enzyme of the respiratory chain, were elevated in the gastrocnemius and soleus muscles of the YBMT mice compared with the corresponding muscles of the control mice (*Table* *S3*). YBMT also improved the levels of PGC‐1α genes in the muscle tissues (*Table* *S3*). Collectively, these findings suggest that in the muscles of SAMP10 mice, the beneficial effects of YBMT are also likely attributable, at least in part, to the amelioration of age‐associated mitochondrial damage and PGC‐1α signalling activation.

### Growth differentiation factor‐11 signalling was required in the young bone marrow transplantation‐mediated skeletal muscle protective actions in senescence‐accelerated mouse prone 10 mice

**Figure 8:**
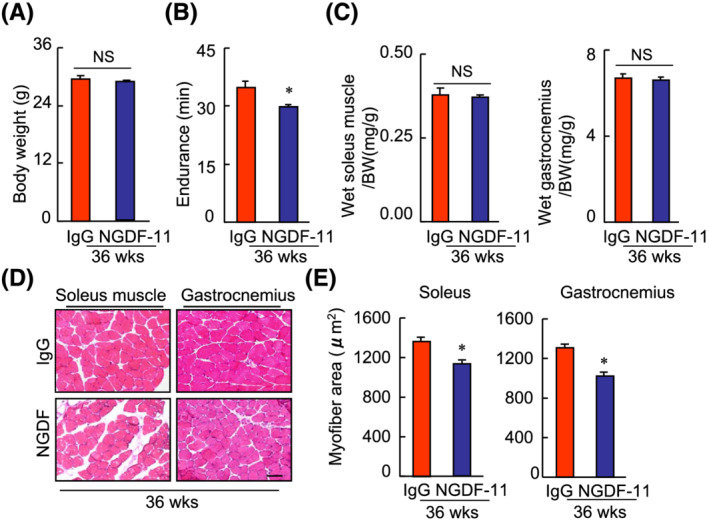
Growth differentiation factor‐11 (GDF‐11) blocking reduced the muscle mass and muscle function of young bone marrow transplantation (YBMT) mice at 32 weeks of age. (*A* and *B*) Body weight (BW) and endurance were recorded in the control (IgG) and YBMT groups. (*C*) The ratios of soleus muscle to BW and the ratios of gastrocnemius to BW were calculated in the two groups. (*D* and *E*) Representative haematoxylin–eosin (H&E) staining images and quantitative data show the myofibre size of gastrocnemius and soleus muscles of both groups. Data are mean ± standard error of the mean (SEM) (*n* = 5–6). *P < 0.05, Student's *t*‐test. Scale bars: 50 μm.

It has been demonstrated that GDF‐11 systemically regulates muscle aging.^21^ The present study's ELISA results revealed that YBMT resulted in increased levels of GDF‐11 (*Table* *S2*). With the exceptions of body weight, the ratio of the soleus muscle to body weight and the ratio of the gastrocnemius muscle to body weight, GDF‐11 blocking reduced not only the endurance capacity of the mice but also the myofibre areas to the soleus and gastrocnemius muscles compared with the corresponding muscles of the IgG‐treated mice that underwent YBMT (*Figure* 8). GDF‐11 depletion decreased the desmin expression and the number of PCNA^+^ proliferating cells and increased the number of TUNEL^+^ apoptotic cells in both muscles (*Figure* *S1*). In contrast, supplementation with rGDF‐11 resulted in increases in the endurance capacity, myofibre area, desmin expression, and proliferating cells and decreased cell apoptosis in both the soleus and gastrocnemius muscles (*Figures* *S2* and *S3*). NGDF‐11 exerted harmful effects on the levels of gp91phox, Bcl‐2, and caspase‐9 proteins in both muscles; and these changes were rectified by rGDF‐11 (*Figure* *S4*). These observations suggest that GDF‐11 signalling is involved in the YBMT‐mediated prevention of muscle atrophy in SAMP10 mice. However, there were no significant differences in the levels of adiponectin, VEGF, bFGF, or TNF‐α between the YBMT and non‐YBMT control mice (*Table* *S2*).

## Discussion

Stem cell therapy‐induced systemic and individual tissue cell responses underlie various benefits. Exploring the potential mechanisms of YBMT‐related modulations of the features of aging‐related muscle diseases will contribute to therapeutic strategies to combat sarcopenia and frailty. The most significant findings of this study are as follows. The SAMP10 mice that underwent YBMT showed an amelioration of aging‐associated changes in the skeletal muscle size and dysfunction after 16 weeks. Here, at the molecular and cellular levels, YBMT elevated not only the plasma GDF‐11 and HDL levels but also the levels of proteins or genes of IRS, p‐Akt, p‐mTOR, myogenin, COX‐4, and PGC‐1α in the soleus and gastrocnemius muscles of SAMP10 mice. Simultaneously, YBMT enhanced the levels of anti‐apoptotic Bcl‐2 protein and lowered the NADPH oxidase gp91phox subunit protein expression, superoxide production, and pro‐apoptotic caspase‐9 protein expression in the tissues of both the soleus and gastrocnemius muscles. Finally, YBMT mice showed enhanced numbers of PCNA^+^, Pax7^+^, and CD34^+^/integrin‐α7^+^ cells and improved the regeneration of muscle. GDF‐11 depletion diminished the YBMT‐mediated muscle benefits, accompanied by harmful molecular changes (in gp91phox, Bcl‐2, and caspase‐9); these changes were rectified by rGDF‐11 treatment.

Cell therapy with BM‐MSCs has often been applied to treat and prevent skeletal muscle atrophy and dysfunction in aged humans and animals.^10^, ^11^, ^12^, ^13^, ^14^ The data obtained in the present study demonstrated that YBMT enhanced the grip strength and endurance of SAMP10 mice. YBMT also elevated the slow HMC‐positive myofibre rate and desmin expression in the gastrocnemius and soleus muscles of the mice. A decline in protein anabolism decline is associated with the skeletal muscle wasting and dysfunction in humans and animals.^5^ Our present findings demonstrated that YBMT increased the numbers of proliferating cells in both types of muscle. The SAMP10 mice that underwent YBMT had elevated levels of protein anabolism‐related investigated molecules, that is, IRS‐1, p‐mTOR, p‐Akt, and p‐ERK1/2. In SAMP10 mice under our experimental conditions, the benefits of YBMT were thus likely to improve muscle integrity via the restoration of the protein anabolism and cell proliferation.

The effect of YBMT on PGC‐1α activity is likely to be of great clinical and experimental significance when the expression of the transcriptional coactivator is impaired.^34^ Accumulating evidence indicates that PGC‐1α expression in skeletal muscle declines with age and with diabetic disease.^4^, ^35^ In the present study, YBMT enhanced the PGC‐1α gene expression. Our findings also show that YBMT mitigated age‐associated mitochondrial injury and lipid droplet accumulation. Our quantitative PCR observations demonstrated that YBMT stimulated the COX4 and PGC‐1α gene expressions. It was reported that PGC‐1α activation accelerated the mitochondrial morphological changes and its dysfunctions in rat muscles.^36^ Based on these findings, we propose that the improvement of the physical performance of SAMP10 mice that underwent YBMT (grip strength and endurance) might be due to the PGC‐1α activation‐mediated mitochondrial biogenesis and energy metabolism. It is also notable that the YBMT in SAMP10 mice resulted in an increase in the plasma metabolic HDL. HDL has been shown to regulate mitochondrial structure and function.^37^ A recent integrative analysis revealed that there is a close association between Tstd1 up‐regulation‐related HDL dysregulation and mitochondrial dysfunction in mice fed a high‐fat diet.^38^ A review article provided novel insights into the role of HDL‐mediated sphingozine‐1‐phosphate in cardiometabolic diseases.^39^ HDL is a stable reservoir of active sphingozine‐1‐phosphate in the circulation.^40^ It was reported that sphingozine‐1‐phosphate can favourably modulate mitochondrial function in damaged cardiomyocytes, at least in part by promoting cytochrome *c* oxidase assembly. Taken together, the past and present findings indicate that an up‐regulation of HDL by YBMT could contribute to the protection of the aging‐related mitochondrial damage and dysfunction in SAMP10 mice. Further studies are needed to explore this issue.

A recent paradigm shift has emerged, suggesting that the beneficial effects of stem cells may not be restricted to cell restoration alone; rather, the effects are also due to their transient paracrine actions.^8^, ^9^, ^10^ GDF‐11 is a secreted factor in the TGF‐β family of cytokines,^41^ and rGDF has been shown to rectify skeletal muscle dysfunction in aged mice.^21^ The ability of YBMT to increase GDF‐11 levels probably contributes to the loss of muscle mass and function in SAMP10 mice. In agreement with a report that cell therapy with BM mitigated endothelial cell apoptosis in ischaemic muscles at advanced ages,^15^ our present results show that cell apoptosis in the soleus and gastrocnemius muscles of the mice were ameliorated by YBMT. The YBMT also significantly enhanced the circulating GDF‐11 levels as well as skeletal muscle morphological changes and dysfunction in SAMP10 mice. YBMT reduced the muscle caspase‐9 level, gp91phox level, and superoxide production and enhanced the Bcl‐2 level in both muscles of the SAMP10 mice, and these changes were diminished by the depletion of GDF‐11. The administration of rGDF‐11 resulted in beneficial skeletal muscle biological and morphological changes as well as improved physical performance in the SAMP10 mice. These data indicate that the up‐regulation of GDF‐11 by YBMT could protect against aging‐associated muscle loss and dysfunction that were mediated, at least in part, through anti‐oxidative stress and anti‐apoptosis in our experimental animal conditions. It should be noted that many GDF11 antibodies also bind GDF‐8 (myostatin) due to their 90% sequence homology in their mature forms.^41^ Further research is necessary to explore whether the binding of a GDF‐11 neutralizing antibody (from R&D Systems in this study) to myostatin alters its biological effect in our experimental mouse model.

Several high‐profile studies reported that GDF‐11 declines with age and that a restoration of systemic GDF‐11 to ‘youthful’ levels is beneficial for several age‐related pathophysiological conditions.^21^, ^22^, ^23^ Aged mice treated with daily intraperitoneal administrations of rGDF‐11 (0.1 mg/kg of body weight) exhibited increased numbers of satellite cells, improved regenerative capacity, and more youthful‐appearing profiles of the myofibre calibre in regenerating muscle compared with control aged mice.^21^ The data presented herein demonstrate that YBMT resulted in increases in the levels of plasma GDF‐11 and the numbers of CD34^+^/integrin‐α7^+^ MuSCs and Pax7^+^ cells in the soleus and gastrocnemius. YBMT also enhanced the myogenin protein expression in both muscles. Cell therapy with BM from GFP^+^‐transgenic mice has resulted in a 4.2–11.4% increase in the number of GFP^+^ myofibres in both muscles' tissues. Our present findings suggest that YBMT restores muscle regeneration and ameliorates dysfunction through its ability to enhance BM‐MSCs' homing and differentiation that were partially mediated by GDF‐11 signalling in aged muscles under our experimental conditions.

However, a comprehensive review article stated that multiple independent research groups sought to better elucidate age‐related changes in circulating GDF‐11 and its function in skeletal muscle regeneration.^41^ It should be noted that there is a discrepancy regarding the potential role of GDF‐11 between present and previous studies.^42^ It was reported that circulating GDF‐11 levels are elevated during aging and that an administration of GDF‐11 disturbed the differentiation and expansion of satellite cells, leading to impaired regenerative capacity.^24^ Bioactive GDF‐11 at supraphysiological levels caused the wasting of both cardiac and skeletal muscles.^22^, ^42^ Our present observations indicate that (*i*) rGDF‐11 may exert anti‐oxidative and anti‐apoptosis effects and mitigate skeletal wasting and (*ii*) NGDF‐11 produced opposite actions. Although we have no direct evidence, the possibility of differences in the models used (e.g. natural aging and senescence‐accelerated aging models) might explain the discrepancies regarding the actions of GDF‐11. Further investigations are needed to determine whether GDF‐11 could be used as a therapeutic agent or is a potentially deleterious biomarker in muscle wasting diseases.

There are several study limitations. First, the SAMP10 model mice are generated by a spontaneous gene mutation and have a short life span (approximately half that of mice in general; approximately 1 year), and they exhibit muscle atrophy and deficits in muscle mass and function. We could not obtain direct evidence regarding the role of GDF‐11 in YBMT‐mediated muscle regeneration and dysfunction in SAMP10 mice. Second, we set the evaluation endpoints as 36 weeks for the rGDF‐11 supplementation and NGDF‐11 supplementation experiments and 40 weeks for the YBMT‐alone experiments. The influence of these different endpoints was not examined. Third, we did not investigate a SAMP10 cardiotoxin‐induced muscle injury model to confirm the YBMT‐mediated recovery of muscle regeneration capacity. Fourth, we have no evidence regarding whether GDF‐11 modulates the differentiation of BM‐MSCs into skeletal muscles *in vivo* and *in vitro*.

## Summary

Cell therapy with BM stimulates the muscles' responses in pathological conditions, including sarcopenia. However, the molecular mechanisms by which BM‐MSCs improve the loss of muscle mass and function that are associated with aging are poorly understood. It was reported that therapies for patients with ischaemic heart disease using stem and progenitor cells from aged patients have been disappointing.^43^ Our present results indicate that in SAMP10 mice, sarcopenia can be ameliorated by YBMT via the improvement of protein anabolic responses, the imbalance between muscle apoptosis and proliferation, and mitochondrial biogenesis that are partially mediated by GDF‐11 signalling. The transplantation of BM from healthy young animals to animals at advanced ages in an attempt to restore the ‘young’ muscle response should be investigated further as a powerful strategy that may prevent age‐associated declines in muscle regeneration and function by recruiting and improving the delivery of BM‐MSCs to the damaged muscle tissues.

## Conflicts of interest

The authors declare that they have no conflicts of interest to disclose with respect to this manuscript.

## Supporting information


**Table S1.** Primer sequences used in the quantitative real‐time PCR
**Table S2.** Levels of plasma investigated growth factors, cytokines and metabolic parameters in two experimental groups at 40 wks.
**Table S3.** Levels of muscle investigated gens in two experimental groups
**Figure S1.** NGDF‐11 produced a harmful effect on desmin expression and cell loss in the soleus and gastrocnemius of YBMT‐mice at 36‐wks of age. (**A**): Fluorescence staining of muscles with laminin 5 rabbit pAb (green) and desmin mAb (red). (**B**): Representative PCNA immunostaining with mouse mAb used to assess the con of proliferated cells. (**C**): Quantitative data showing for PCNA^+^ cells in both muscles. (**D**): Representative TUNEL staining used to assess the content of apoptotic cells. (**E**): Quantitative data show TUNEL+ cells in both muscles. Data are means ± SEM (*n* = 5–7). *p < 0.05, **p < 0.01 vs. the corresponding control groups by one‐way ANOVA followed by Tukey post hoc tests. Arrowhead: related staining positive cells. Scale bars: 50 μm.
**Figure S2.** Administration of rGDF‐11 improved muscle mass and muscle function in YBMT mice at 36‐wks of age. (**A**,**B**): Body weight and endurance were recorded in the control (IgG) and YBMT groups. (**C**): Ratios of soleus muscle to BW and ratios of gastrocnemius to BW were calculated in the two groups. (**D**,**E**): Representative images and quantitative data show the myofiber size of gastrocnemius and soleus muscles of both groups. Data are means ± SEM (*n* = 5–6). Significance was estimated using Student's *t*‐test (*p < 0.05). Scale bars: 50 μm.
**Figure S3.** rGDF‐11 produced a beneficial effect on desmin expression and cell loss in the soleus and gastrocnemius of YBMT‐mice at 36‐wks of age. (**A**): Fluorescence staining of muscles with laminin 5 rabbit pAb (green) and desmin mAb (red). (**B**): Representative PCNA immunostaining with mouse mAb used to assess the con of 6 proliferated cells. (**C**): Quantitative data showing for PCNA^+^ cells in both muscles. (**D**): Representative TUNEL staining used to assess the content of apoptotic cells. (**E**): Quantitative data show TUNEL+ cells in both muscles. Data are means ± SEM (*n* = 5–6). *p < 0.05, **p < 0.01 vs. the corresponding control groups by one‐way ANOVA followed by Tukey post hoc tests. Arrowhead: related staining positive cells. Scale bars: 50 μm.
**Figure S4.** The effects of NGDF‐11 or rGDF‐11 on the investigated proteins in the muscles of two experimental groups at 36 wks of age. A–D: Representative immunoblotting images and combined quantitative data of the levels of gp91phox, Bcl‐2, and caspase‐9 in the soleus and gastrocnemius muscles of the IgG and NGDF‐11 groups (**A**,**B**) and saline and rGDF‐11 groups (**C**,**D**). Data are mean ± SEM (*n* = 5–6). *p < 0.05, Student's *t*‐test.
