# Supplementary material for: Young bone marrow transplantation prevents aging‐related muscle atrophy in a senescence‐accelerated mouse prone 10 model
Source: J Cachexia Sarcopenia Muscle. 2022 Sep 4;13(6):3078–90. doi: 10.1002/jcsm.13058 (PMC9745469; doi:10.1002/jcsm.13058)
Supplement: Supplementary file 1 — Table S1. Primer sequences used in the quantitative real‐time PCR Table S2. Levels of plasma investigated growth factors, cytokines and metabolic parameters in two experimental groups at 40 wks. Table S3. Levels of muscle investigated gens in two experimental groups Figure S1. NGDF‐11 produced a harmful effect on desmin expression and cell loss in the soleus and gastrocnemius of YBMT‐mice at 36‐wks of age. (A): Fluorescence staining of muscles with laminin 5 rabbit pAb (green) and desmin mAb (red). (B): Representative PCNA immunostaining with mouse mAb used to assess the con of proliferated cells. (C): Quantitative data showing for PCNA+ cells in both muscles. (D): Representative TUNEL staining used to assess the content of apoptotic cells. (E): Quantitative data show TUNEL+ cells in both muscles. Data are means ± SEM (n = 5–7). *p < 0.05, **p < 0.01 vs. the corresponding control groups by one‐way ANOVA followed by Tukey post hoc tests. Arrowhead: related staining positive cells. Scale bars: 50 μm. Figure S2. Administration of rGDF‐11 improved muscle mass and muscle function in YBMT mice at 36‐wks of age. (A,B): Body weight and endurance were recorded in the control (IgG) and YBMT groups. (C): Ratios of soleus muscle to BW and ratios of gastrocnemius to BW were calculated in the two groups. (D,E): Representative images and quantitative data show the myofiber size of gastrocnemius and soleus muscles of both groups. Data are means ± SEM (n = 5–6). Significance was estimated using Student's t‐test (*p < 0.05). Scale bars: 50 μm. Figure S3. rGDF‐11 produced a beneficial effect on desmin expression and cell loss in the soleus and gastrocnemius of YBMT‐mice at 36‐wks of age. (A): Fluorescence staining of muscles with laminin 5 rabbit pAb (green) and desmin mAb (red). (B): Representative PCNA immunostaining with mouse mAb used to assess the con of 6 proliferated cells. (C): Quantitative data showing for PCNA+ cells in both muscles. (D): Representative TUNEL staining used t [file JCSM-13-3078-s001.pdf]

Supplementary Data

Young bone marrow transplantation prevents aging-related muscle atrophy in a senescence-accelerated mouse prone 10 model

Corresponding Author: Dr. Xian Wu Cheng

Dr. Xian Wu Cheng, Department of Cardiology and Hypertension, Yanbian University Hospital, 1327 Juzijie, Yanji, Jilin 133000, China. Tel: +86-433-2660131; Fax: +86-433-2513610

Table S1. Primer sequences used in the quantitative real-time PCR

Gene	Forward primer (5' to 3')	Reverse primer (5' to 3')	GenBank no.
COX-III	TCTTCATGGCTACTGGATTCCA	ATCATGCTGCGGCTTCAAAT	NC_005089
COX-IV	AGCTGAGCCAAGCAGAGAAG	AATCACCAGAGCCGTGAATC	NM_053091
GLUT-4	GACGGACACTCCATCTGTTG	GCCACGATGGAGACATAGC	NM_009204
PGC-1α	CCGAGAATTCATGGAGCAAT	TTTCTGTGGGTTTGGTGTGA	NM_008904
PGC-1β	ACGGTTTTATCACCTTCCGG	ATAGCTCAGGTGGAAGGAGGG	NM_133249
GAPDH	ATGTGTCCGTCGTGGATCTGA	ATGCCTGCTTCACCACCTTCT	NM_008084

Abbreviations: COX-III: cytochrome *c* oxidase-III; COX-IV: cytochrome *c* oxidase-IV; GLUT-4, glucose transporter-4; PGC-1α, peroxisome proliferator-activated receptor-γ coactivator-1α; GAPDH: glyceraldehyde 3-phosphate dehydrogenase.

Table S2. Levels of plasma investigated growth factors, cytokines and metabolic parameters in two experimental groups at 40 wks.

Parameters	Control	YBMT
T-cho (mg/dL)	68.9 ± 5.5	60.2± 4.0
LDL (mg/dL)	3.48 ± 1.1	4.4 ± 0.5
HDL (mg/dL)	9.4 ± 1.8	15.7 ± 1.7*
Triglyceride	45.6 ± 10.8	28.0 ± 5.6
Glucose (mg/dL)	115.3 ± 8.7	118.9 ± 4.2
BUN (mg/dL)	27.3 ± 1.4	28.2 ± 1.0
Cre (mg/dL)	0.06 ± 0.01	0.09 ± 0.01
TNF-α (pg/mL)	96.6 ± 9.6	91.9 ± 8.5
Adiponectin (ng/mL)	1052 ± 22	1080 ± 16
VEGF (pg/mL)	29.2 ± 3.4	34.3 ± 2.8
bFGF (pg/mL)	71.2 ± 3.8	83.7 ± 4.9
GDF-11 (pg/mL)	27.9 ± 4.2	64.6 ± 9.9*

Values are expressed as mean ± SEM. TNF-α, tumor necrosis-factor-α; VEGF, vascular endothelial growth factor; GDF-11, growth differentiation factor; T-cho, total cholesterol; LDL, low-density lipoprotein; HDL, high-density lipoprotein; TG, triglyceride; BUN, Blood urea nitrogen; Cre, creatinine. *$P < 0.05$ compared with control group (Non-YBMT).

Table S3. Levels of muscle investigated gens in two experimental groups

RT-PCR(Soleus muscle)	Control	YBMT
COX4	22.8 ± 2.8	84.7 ± 13.7**
COX3	68.1 ± 10.9	117.2 ± 29.1
GLUT4	6.0 ± 2.0	9.3 ± 4.9
PGC-1α	7.9 ± 1.4	45.9 ± 16.2*
RT-PCR(Gastrocnemius muscle)		
COX4	21.7 ± 4.4	37.0 ± 11.3*
COX3	10.1 ± 3.8	60.6 ± 26.6
GLUT4	44.9 ± 15.9	50.0 ± 19.3
PGC-1α	18.7 ± 4.1	47.2 ± 11.4*

Values are expressed as mean ± SEM. *P < 0.05 compared with control (Non-BMCT) value. †P < 0.05 compared with control group.

Supplementary Figure Legends

Figure S1. NGDF-11 produced a harmful effect on desmin expression and cell loss in the soleus and gastrocnemius of YBMT-mice at 36-wks of age. (**A**): Fluorescence staining of muscles with laminin 5 rabbit pAb (green) and desmin mAb (red). (**B**): Representative PCNA immunostaining with mouse mAb used to assess the con of proliferated cells. (**C**): Quantitative data showing for $PCNA^+$ cells in both muscles. (**D**): Representative TUNEL staining used to assess the content of apoptotic cells. (**E**): Quantitative data show $TUNEL^+$ cells in both muscles. Data are means ± SEM (n=5–7). *$p<0.05$, **$p<0.01$ vs. the corresponding control groups by one-way ANOVA followed by Tukey post hoc tests. Arrowhead: related staining positive cells. Scale bars: 50 µm.

Figure S2. Administration of rGDF-11 improved muscle mass and muscle function in YBMT mice at 36-wks of age. (**A**,**B**): Body weight and endurance were recorded in the control (IgG) and YBMT groups. (**C**): Ratios of soleus muscle to BW and ratios of gastrocnemius to BW were calculated in the two groups. (**D**,**E**): Representative images and quantitative data show the myofiber size of gastrocnemius and soleus muscles of both groups. Data are means ± SEM (n=5–6). Significance was estimated using Student's *t*-test (*$p<0.05$). Scale bars: 50 µm.

Figure S3. rGDF-11 produced a beneficial effect on desmin expression and cell loss in the soleus and gastrocnemius of YBMT-mice at 36-wks of age. (**A**): Fluorescence staining of muscles with laminin 5 rabbit pAb (green) and desmin mAb (red). (**B**): Representative PCNA immunostaining with mouse mAb used to assess the con of

proliferated cells. (**C**): Quantitative data showing for $PCNA^+$ cells in both muscles. (**D**): Representative TUNEL staining used to assess the content of apoptotic cells. (**E**): Quantitative data show $TUNEL^+$ cells in both muscles. Data are means ± SEM (n=5–6). **p*<0.05, ***p*<0.01 vs. the corresponding control groups by one-way ANOVA followed by Tukey post hoc tests. Arrowhead: related staining positive cells. Scale bars: 50 µm.

Figure S4. The effects of NGDF-11 or rGDF-11 on the investigated proteins in the muscles of two experimental groups at 36 wks of age. A–D: Representative immunoblotting images and combined quantitative data of the levels of gp91phox, Bcl-2, and caspase-9 in the soleus and gastrocnemius muscles of the IgG and NGDF-11 groups (**A**,**B**) and saline and rGDF-11 groups (**C**,**D**). Data are mean ± SEM (n=5–6). *p<0.05, Student's *t*-test.

Figure S1

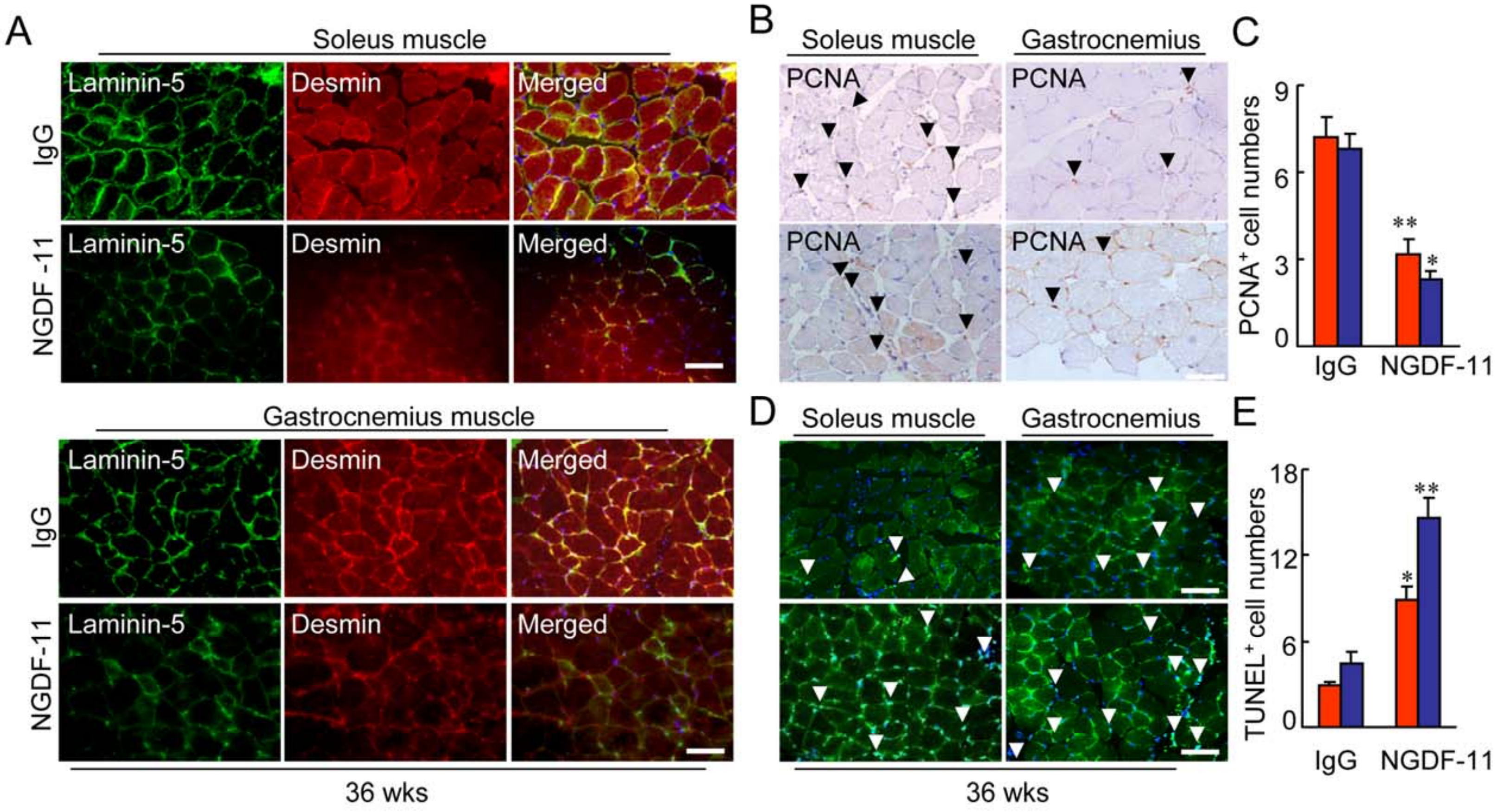
A
Soleus muscle
Laminin-5
Desmin
Merged
IgG
NGDF -11
Gastrocnemius muscle
NGDF-11
36 wks
B
Soleus muscle
Gastrocnemius
PCNA
C
PCNA+ cell numbers
9
6
3
0
IgG
NGDF-11
**
*
D
E
TUNEL+ cell numbers
18
12
6
0
36 wks

Figure S2

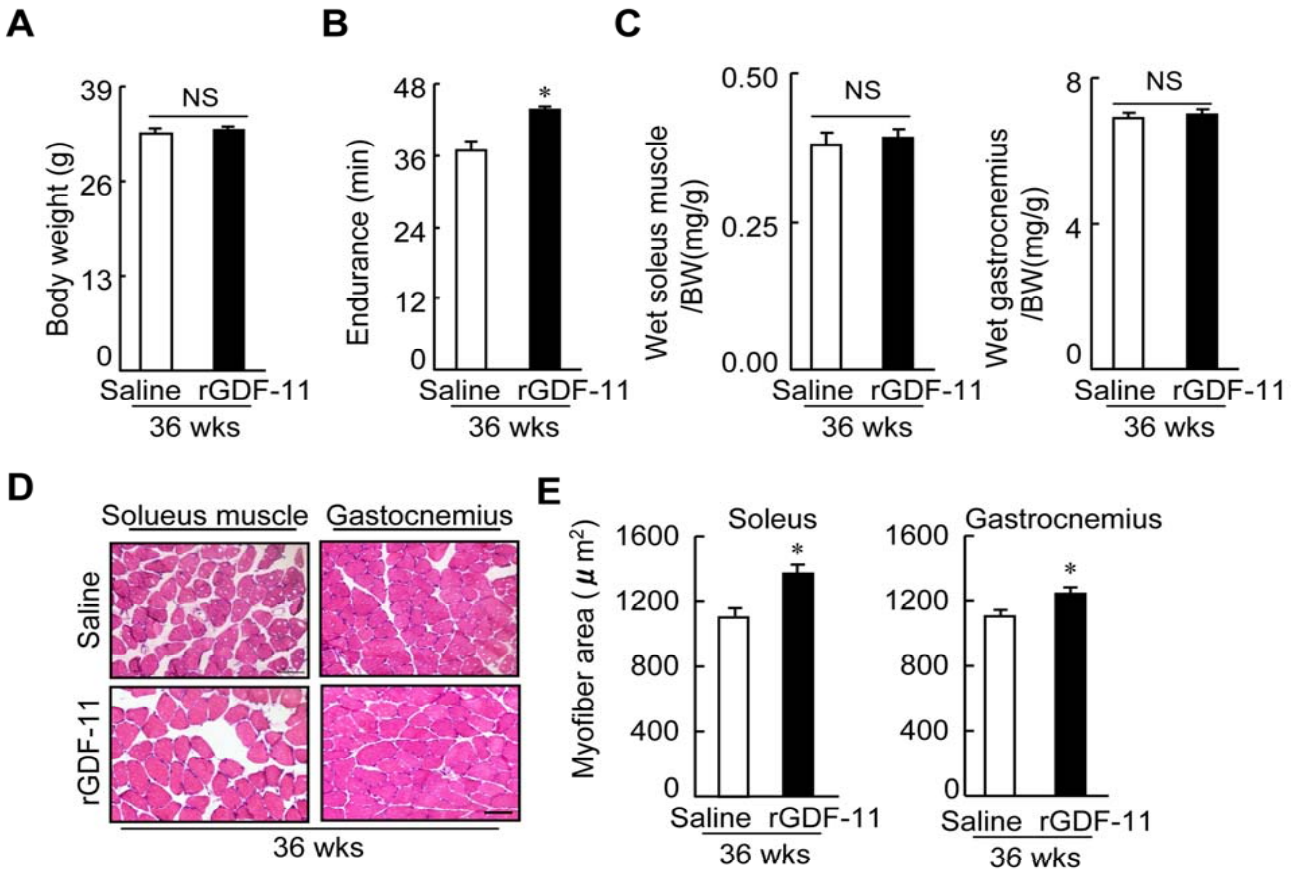

A
B
C
39
26
13
0
Body weight (g)
NS
Saline rGDF-11
36 wks
48
36
24
12
0
Endurance (min)
*
Saline rGDF-11
36 wks
0.50
0.25
0.00
Wet soleus muscle /BW(mg/g)
NS
Saline rGDF-11
36 wks
8
4
0
Wet gastrocnemius /BW(mg/g)
NS
Saline rGDF-11
36 wks
D
Solueus muscle
Gastocnemius
Saline
rGDF-11
36 wks
E
Soleus
Gastrocnemius
1600
1200
800
400
0
Myofiber area (μm2)
*
Saline rGDF-11
36 wks

Figure S3

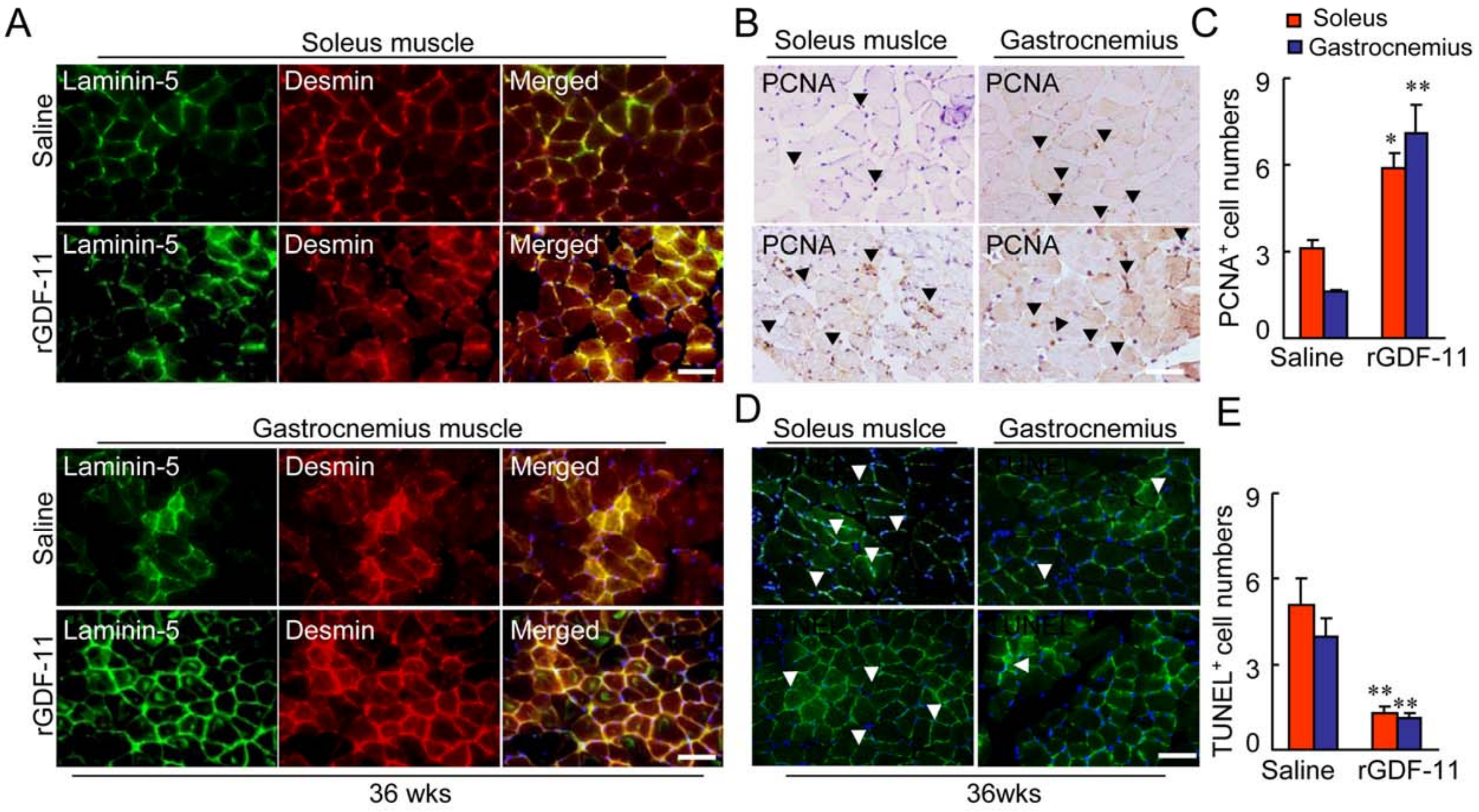
A
Soleus muscle
Saline
rGDF-11
Laminin-5
Desmin
Merged
Gastrocnemius muscle
36 wks
B
Soleus muslce
Gastrocnemius
PCNA
C
Soleus
Gastrocnemius
PCNA⁺ cell numbers
Saline
rGDF-11
D
Soleus muslce
Gastrocnemius
36wks
E
TUNEL⁺ cell numbers
Saline
rGDF-11

Figure S4

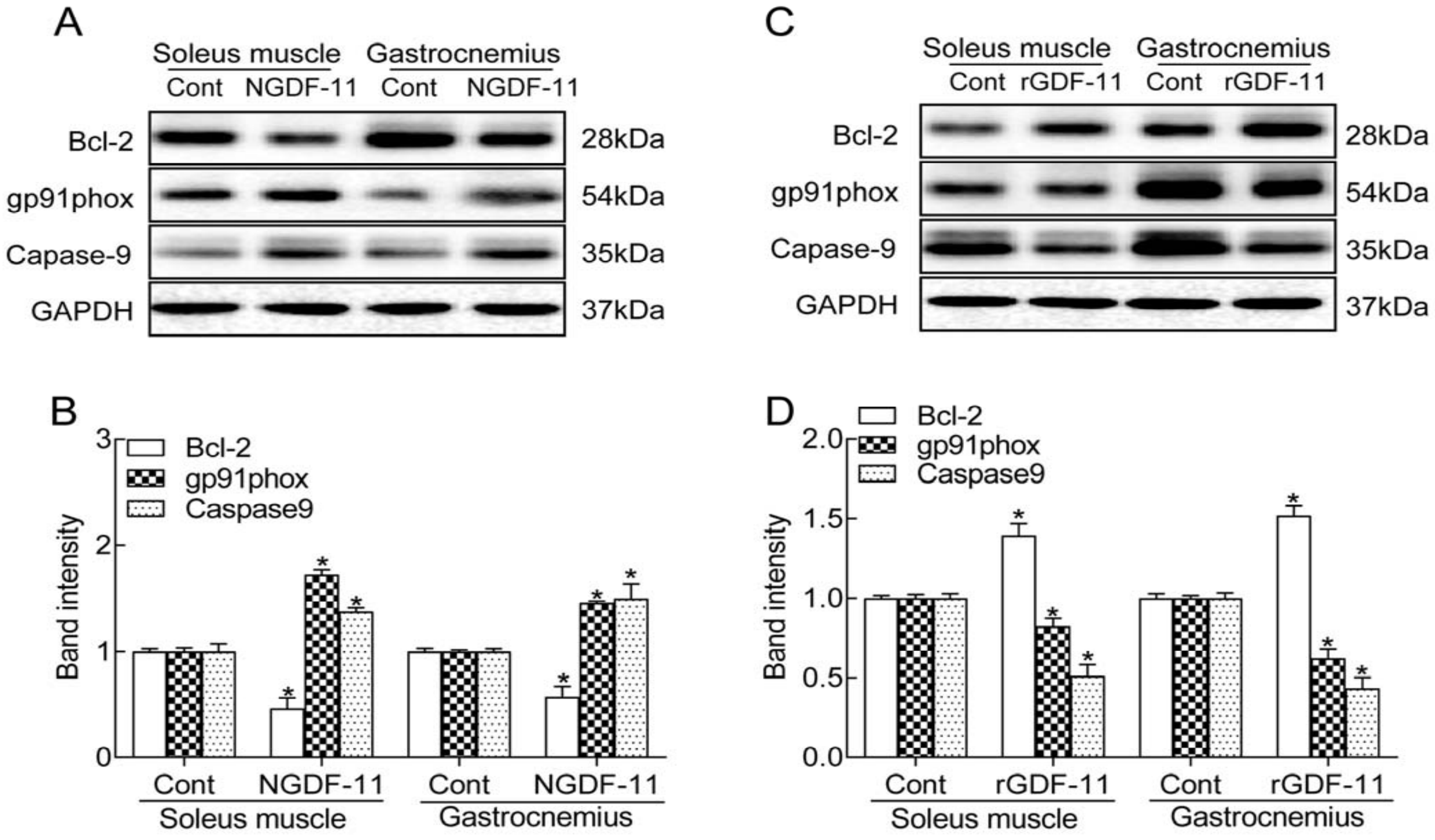
A
Soleus muscle
Gastrocnemius
Cont
NGDF-11
Cont
NGDF-11
Bcl-2
gp91phox
Capase-9
GAPDH
28kDa
54kDa
35kDa
37kDa
C
Soleus muscle
Gastrocnemius
Cont
rGDF-11
Cont
rGDF-11
Bcl-2
gp91phox
Capase-9
GAPDH
28kDa
54kDa
35kDa
37kDa
B
Bcl-2
gp91phox
Caspase9
Band intensity
Cont
NGDF-11
Cont
NGDF-11
Soleus muscle
Gastrocnemius
D
Bcl-2
gp91phox
Caspase9
Band intensity
Cont
rGDF-11
Cont
rGDF-11
Soleus muscle
Gastrocnemius
